# A scalable human iPSC-based neuromuscular disease model on suspended biobased elastomer nanofiber scaffolds

**DOI:** 10.1088/1758-5090/acf39e

**Published:** 2023-09-05

**Authors:** Aimee Cheesbrough, Peter Harley, Federica Riccio, Lei Wu, Wenhui Song, Ivo Lieberam

**Affiliations:** 1 Centre for Gene Therapy & Regenerative Medicine, Faculty of Life Sciences & Medicine, King’s College London, London SE1 9RT, United Kingdom; 2 Centre for Developmental Neurobiology, Institute of Psychiatry, Psychology and Neuroscience, King’s College London, London SE1 1UL, United Kingdom; 3 UCL Centre for Biomaterials in Surgical Reconstruction and Regeneration, Department of Surgical Biotechnology, Division of Surgery and Interventional Science, University College London, London NW3 2PF, United Kingdom

**Keywords:** scalable neuromuscular disease modelling, biobased elastomer nanofibers, high content image analysis, human induced pluripotent stem cells, CRISPR-Cas9, optogenetics, amyotrophic lateral sclerosis

## Abstract

Many devastating neuromuscular diseases currently lack effective treatments. This is in part due to a lack of drug discovery platforms capable of assessing complex human neuromuscular disease phenotypes in a scalable manner. A major obstacle has been generating scaffolds to stabilise mature contractile myofibers in a multi-well assay format amenable to high content image (HCI) analysis. This study describes the development of a scalable human induced pluripotent stem cell (iPSC)-neuromuscular disease model, whereby suspended elastomer nanofibers support long-term stability, alignment, maturation, and repeated contractions of iPSC-myofibers, innervated by iPSC-motor neurons in 96-well assay plates. In this platform, optogenetic stimulation of the motor neurons elicits robust myofiber-contractions, providing a functional readout of neuromuscular transmission. Additionally, HCI analysis provides rapid and automated quantification of axonal outgrowth, myofiber morphology, and neuromuscular synapse number and morphology. By incorporating amyotrophic lateral sclerosis (ALS)-related TDP-43^G298S^ mutant motor neurons and CRISPR-corrected controls, key neuromuscular disease phenotypes are recapitulated, including weaker myofiber contractions, reduced axonal outgrowth, and reduced number of neuromuscular synapses. Treatment with a candidate ALS drug, the receptor-interacting protein kinase-1 (RIPK1)-inhibitor necrostatin-1, rescues these phenotypes in a dose-dependent manner, highlighting the potential of this platform to screen novel treatments for neuromuscular diseases.

## 1. Introduction

Neuromuscular diseases represent a diverse class of disorders with unmet clinical need. In several diseases such as amyotrophic lateral sclerosis (ALS) and spinal muscular atrophy (SMA) degeneration of motor neurons, the nerve cells that innervate skeletal muscle, leads to progressive paralysis and death [1, 2]. Conversely, in muscular dystrophies, deterioration of neuromuscular function is caused by progressive weakness and wasting of the muscle [3]. Furthermore, in several autoimmune disorders, such as myasthenia gravis, degeneration of the neuromuscular junction (NMJ) itself, triggered by an autoimmune attack, leads to impaired movement [4]. In many of these conditions peripheral axonal and synaptic dysfunction are key pathological events. Indeed, ALS is characterised by early degeneration of peripheral motor axons and neuromuscular synapses prior to cell death within the central nervous system and progressive paralysis [1, 5]. Currently, there is no cure for this disease, yet growing evidence suggests that preserving neuromuscular synapses can extend lifespan in animal models and in patients [6, 7].

2D co-culture systems of primary motor neurons and myofibers which model nerve-muscle connectivity were first established in the 1970s [8], and later adapted to neuromuscular circuits containing mouse [9] and human [10] pluripotent stem cell-derived motor neurons. However, a major hurdle for applying such co-cultures to developing new treatments for axonal and synaptic degeneration in neuromuscular diseases is the lack of scalable *in vitro* disease models, amenable to high-throughput screening, that recapitulate complex disease phenotypes. A significant challenge has been stabilising mature contractile myofibers in a multi-well format that is suitable for automated high content image (HCI) analysis. In 2D cultures, contractile myofibers detach from the rigid tissue culture plate surface, precluding longitudinal phenotypic analysis [11, 12]. Several 3D solutions to this problem have involved suspending bundles of myofibers between flexible micropillars [13–17] or nylon hooks of Velcro^™^ fabric [18], or attaching a hydrogel-embedded sheet of myofibers to flat polymer anchor points within compartmentalised tissue culture devices [19–21], but the scalability of these approaches and amenability to automated HCI analysis has been limited. Nanofiber scaffolds have been used to successfully stabilise cardiomyocytes in multi-well assay plates [22], however, in this instance, rigid attachment of the nanofibers to the tissue-culture surface precludes elastic recoil of the nanofibers upon muscle contraction, making this approach less suitable for culturing skeletal myofibers. Finally, while a number of approaches have focussed on generating scalable myogenic screening platforms in multi-well formats [16, 17, 23], to our knowledge no neuromuscular co-culture platform has previously been generated in a 96-well assay format compatible with existing HCI analysis platforms.

This study describes the development of a scalable and stable 96-well human induced pluripotent stem cell (hiPSC) neuromuscular disease model, amenable to automated HCI analysis, following our previous study on the growth of hiPSC-myofibers on elastomer nanofibers [24]. To achieve this, suspended and uniformly-aligned elastomer nanofiber sheets are manufactured on custom-built 96-well plates in order to support the formation, contraction, stability and maturation of functional neuromuscular circuits. Optogenetic stimulation of the motor neurons induced robust myofiber contractions, providing a functional readout of neuromuscular transmission. By incorporating ALS-related hiPSC-motor neurons and CRISPR corrected controls it was possible to demonstrate the feasibility of this approach for neuromuscular disease modelling and drug discovery.

## 2. Results

### 2.1. Generation of scalable 96-well hiPSC neuromuscular co-cultures on biobased elastomer nanofibers

**Figure 1.**
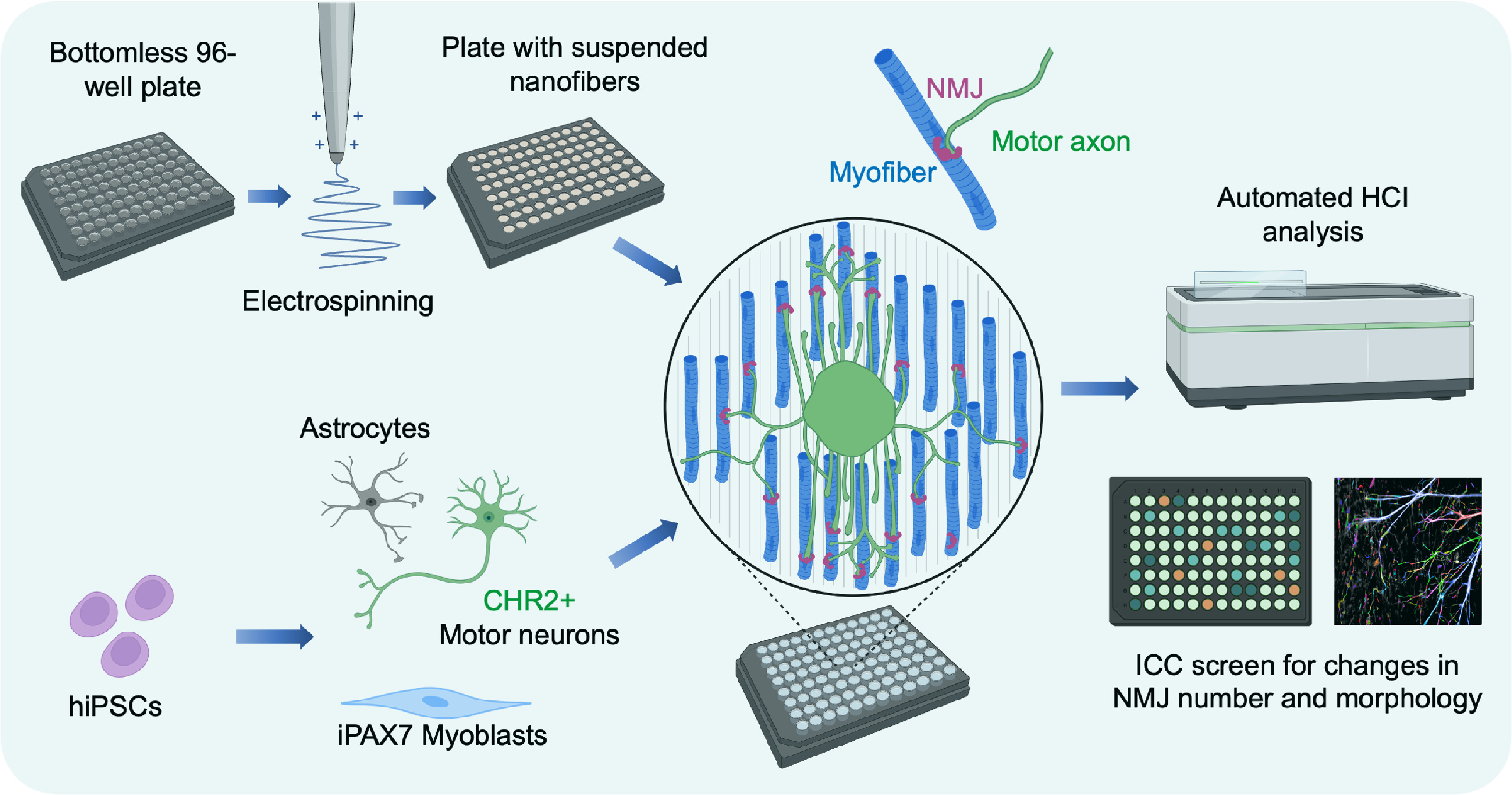
Conceptual schematic of suspended biobased elastomer nanofibers for supporting neuromuscular co-cultures in a scalable 96-well plate format. Optogenetically controlled motor neurons are derived from hiPSCs and made to form neural spheroids with mESC-derived astrocytes. Optogenetically controllable neural spheroids are then plated on a sheet of skeletal myofibers, derived from hiPSCs using inducible PAX7 forward programming. Aligned, suspended nanofibers guide the maturation of the neuromuscular co-cultures and stabilise myofiber contractility, promoting long term survival and maturation in 96-well imaging plates. The standardised plate format, compatible with commercial HCI analysis systems, enables automated HCI analysis of the neuromuscular co-cultures, such that changes in NMJ number and morphology can be screened.

**Figure 2.**
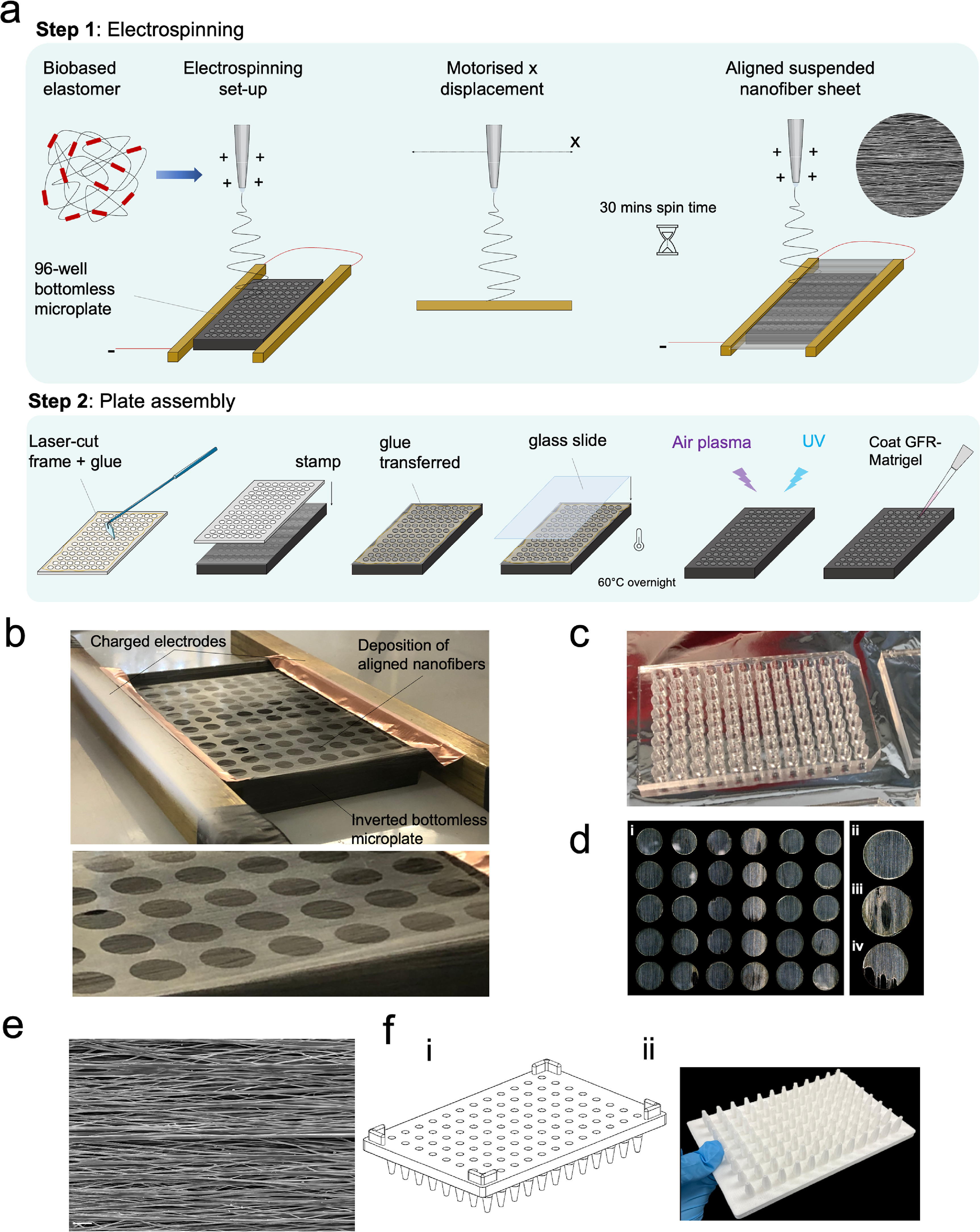
Manufacturing suspended biobased elastomer nanofiber scaffolds in 96-well imaging plates. (a) Schematic outlining the manufacturing process. In step 1 elastomer nanofibers are electrospun onto bottomless 96-well microplates; insert: morphology of nanofibers by scanning electron microscopy (SEM). In step 2 glass bottoms are attached to the plates, plasma treated and coated with GFR-Matrigel. (b) Manufacture of custom built 96-well plates. Aligned nanofibers deposited on bottomless-96-well plate between two charged electrodes. (c) Acrylic stamp used to glue glass base to plate. (d) (i) Examples of different nanofiber wells in a 96-well plate. Most wells had uniform alignment (ii), whilst some, typically at the edges of the plate, had minor or major defects ((iii) & (iv)) Wells were inspected before use and only uniform wells were seeded with cells. (e) SEM image of aligned P(EDS)UU-POSS nanofibers. Scale bar: 10 *µ*m (f) (i) CAD model and (ii) 3D printed seeding mask used to rapidly seed neural aggregates at the centre of each elastomer nanofiber well.

Based on our previously published work on generating aligned elastomer nanofibers to support *in vitro* hiPSC-derived skeletal myofibers [24], we designed and scaled up the device, and further developed neuromuscular co-cultures in high-throughput 96-well assay plates (figure 1). To achieve this, a bottomless standard 96-well plate, with dimensions (length × width) of 127 mm × 86 mm, was placed between electrodes and well-aligned nanofibers were electrospun across the plate, for 30 min, by a moving spinneret needle controlled by a step-motor between two collecting electrodes (figures 2(a) and (b)). A glass base with the same dimensions as the 96-well plate was adhered to the plate using a custom laser-cut acrylic stamp and cured at 60 °C overnight (figures 2(a) and (c)). Nanofibers were deposited uniformly across each well with minimal defects (figure 2(d))—75% of all wells showed an even distribution of aligned nanofibers and were deemed suitable for the co-cultures assay (figure 2(d)(ii)–(iv), supplementary figure S1). A scanning electron microscopy (SEM) image of an aligned nanofiber sheet is shown in figure 2(e). The diameter and alignment of the aligned nanofibers, their morphology and mechanical properties were characterised previously [24]. In preparation for cell culture, plates were plasma-treated, UV sterilised and coated with growth-factor-reduced (GFR)-Matrigel overnight (figure 2(a)). In addition to this we designed and 3D-printed a seeding mask to ensure uniform placement of neural aggregates in the centre of the wells (figure 2(f)).

**Figure 3.**
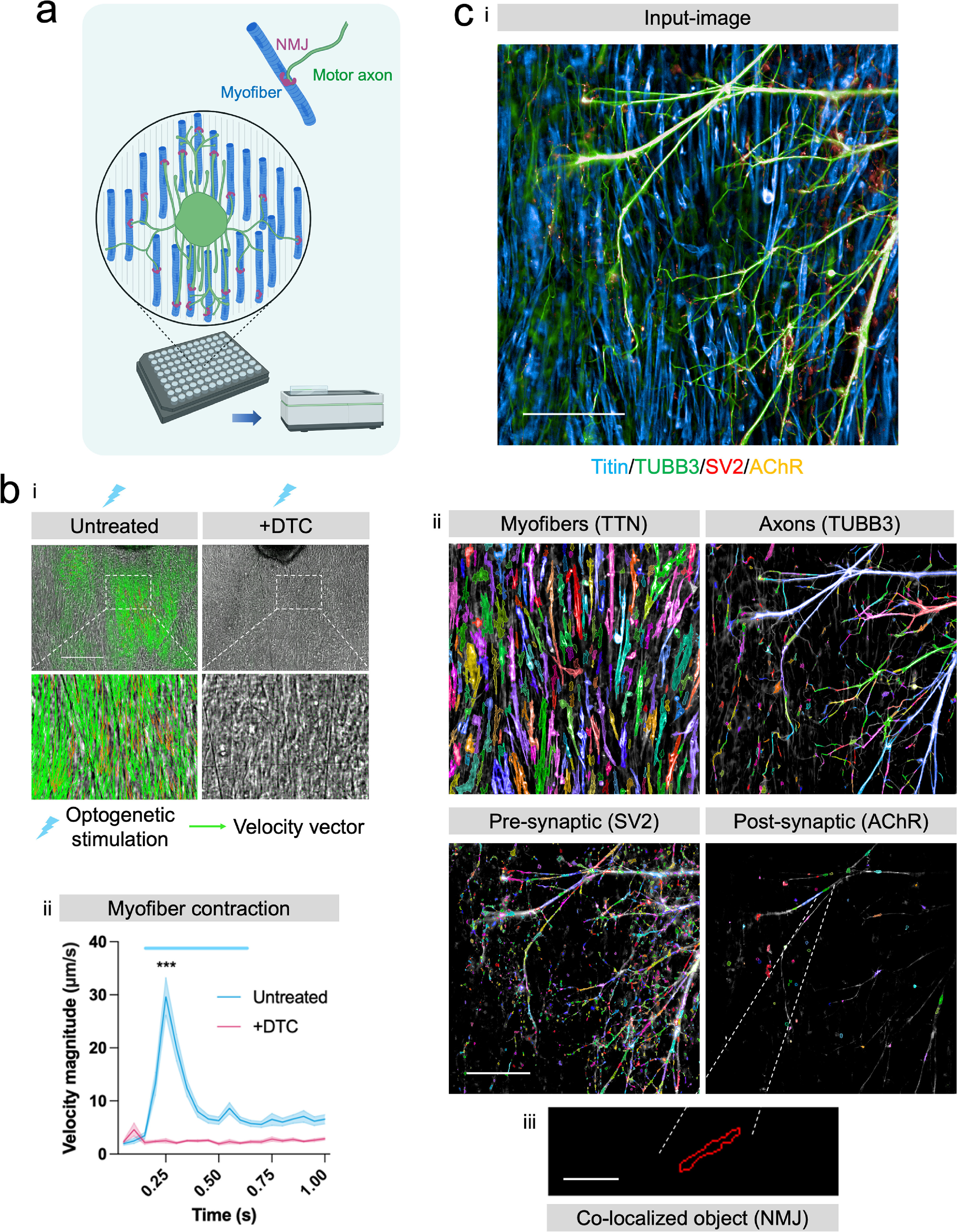
Generation of functional hiPSC-derived neuromuscular co-cultures on suspended biobased elastomer nanofiber scaffolds in 96-well plates. (a) Schematic outlining the format of the neuromuscular co-cultures, whereby optogenetically controllable motor neuron/astrocyte spheroids are plated on top of a sheet of hiPSC-myofibers on the suspended elastomer nanofibers. (b) (i) Optogenetic stimulation of the motor neurons elicits robust myofiber contractions (*n* = 8 wells) that can be blocked by the AChR blocker d-Tubocurarine (DTC) (*n* = 8 wells). One representative of three experiments is shown. Green arrows indicate velocity vectors. Scale bar = 200 *μ*m (top row). (ii) Contractions were quantified using PIV analysis. (c) (i) Single field image taken of mature neuromuscular co-cultures using an automated Operetta CLS HCI system after 2 weeks of culture. The input image shown here to illustrate HCI analysis is the same as in figure 5(c) (corrected genotype). Scale bar = 200 *μ*m (ii) Automatically generated masks used to quantify myofibers (TTN), axons (TUBB3), pre-synaptic (SV2) and post-synaptic (AChR) objects. Scale bar = 200 *μ*m. (iii), Colocalization of pre- and post-synaptic objects defines the NMJ objects. Scale bar = 20 *μ*m. Error bars represent the SEM, unpaired non-parametric t-test used to ascertain statistical significance. ***p < 0.001.

Subsequently, hiPSC-derived myoblasts (4 × 10^4^ cells per well) were seeded onto the elastomer nanofibers and grown for 3 d. Myofibers were derived by forward programming of hiPSCs carrying a dox-inducible *PAX7* transgene [24, 25]. One neural aggregate per well, comprising of 1 × 10^4^ magnetic-activated cell sorting (MACS)-enriched hiPSC-motor neurons suitable for optogenetics (ChR2-YFP^+^) (supplementary figure S2), and 5 × 10^3^ GDNF-expressing mouse embryonic stem cell (mESC)-derived astrocytes was seeded on top of the hiPSC-myofibers (figure 3(a), supplementary figure S3). The custom seeding mask (figure 2(f)) ensured aggregates were deposited in the centre of each well in a rapid manner. After 2 weeks of co-culture, functional NMJs had formed between the hiPSC motor neurons and myofibers. This was evidenced by the observation that optogenetic activation of the motor neurons could elicit robust myofiber contractions, quantifiable using particle image velocimetry (PIV) analysis (figure 3(b)). Blocking acetylcholine receptors (AChR) using d-tubocurarine (DTC) abolished neuromuscular transmission and optogenetically evoked myofiber contractions (figure 3(b)). Automated HCI of the entire plate was performed on the co-cultures using an Operetta CLS HCI analysis system (figure 3(c), supplementary figure S4). Titin staining was used to generate myofiber masks, TUBB3 staining used to generate motor axon masks, SV2 staining used to generate pre-synaptic masks and AChR staining used to generate post-synaptic masks (figure 3(c)(ii)). Co-localisation of pre- and post-synaptic structures was used to derive neuromuscular synapse parameters (figure 3(c)(iii)). Additional filtering based on fluorescence intensity and sphericity was used to eliminate background noise and debris from the analysis.

### 2.2. Nanofiber elastomers support alignment, contraction, long-term stability, and maturation of 96-well neuromuscular co-cultures

**Figure 4.**
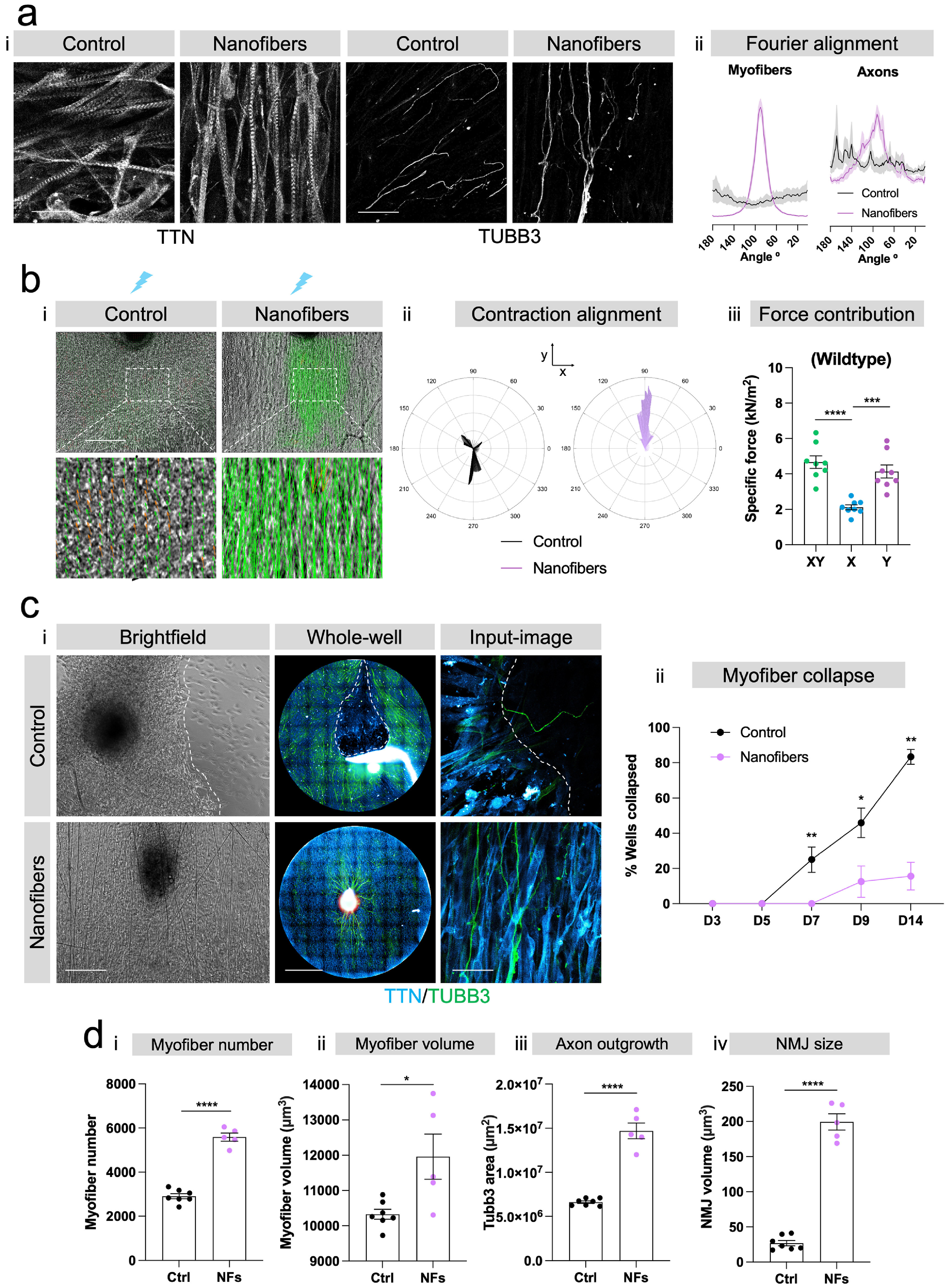
Elastomer nanofibers support alignment, contraction, long-term stability, and maturation of 96-well hiPSC neuromuscular co-cultures at the 2 weeks timepoint. (a) Myofiber and axonal alignment on control (*n* = 8) and nanofiber (*n* = 8) plates. (i) Immunofluorescence images of TTN and TUBB3 staining (scale bar = 50 *μ*m). (ii) Alignment quantified using Fourier transformation (*n* = 8 wells). (b) (i) Myofiber contraction direction based on PIV analysis. (ii) Compass plots show angle of contraction against velocity. (iii) Calculated peak *XY, Y*-specific and *X*-specific forces from PIV velocity values and comparison of *XY, Y* and *X* axis contribution to muscle contraction force (*n* = 8 culture wells). (c) (i) Brightfield, whole well and single field of view images showing examples of collapsed myofiber sheets on control plates vs intact sheets on nanofiber plates. Scale bars: 200 *μ*m, 2500 *μ*m, 50 *μ*m. (ii) Quantification of myofiber collapse overtime on control vs nanofiber plates (combined data from three independent experiments; *n* = 8 wells per experiment). (d) Quantification of neuromuscular parameters: (i) myofiber number, (ii) myofiber volume, (iii) axon outgrowth and (iv) NMJ size on control vs nanofiber plates (control: *n* = 7, nanofibers: *n* = 5 wells). Error bars represent the SEM, unpaired non-parametric t-test used to ascertain statistical significance. *p< 0.05, **p < 0.01, ***p < 0.001, ****p < 0.0001.

Having established hiPSC-derived neuromuscular co-cultures on elastomer nanofibers in 96-well plates we next wanted to assess whether the aligned elastomer nanofibers supported myofiber alignment and contraction, as well as long-term stability and maturation of the co-cultures compared to control plates with no nanofibers. We found that nanofibers drastically improved myofiber alignment (figures 4(a) and (b)), consistent with our recent work [17]. This resembles the parallel alignment of myofibers in *in vivo* muscle, which is crucial for fusion and maturation. We also noted that nanofibers improved axonal alignment and outgrowth (figures 4(a)(ii) and (d)(iii)). The coordination of both aligned axons and myofibers subsequently caused a uniform directionality of optogenetically evoked myofiber contractions (figure 4(b)(i), (ii)). This was further evidenced by the fact that the specific contraction force contribution came largely from the *Y*-axis—the axis of nanofiber alignment (figure 4(b)(iii)). Nanofibers also supported long-term neuromuscular co-cultures: In control plates without nanofiber scaffolds, myofiber collapse was a common issue after only 7 d in culture, and present in nearly all wells after 2 weeks in culture (figure 4(c), supplementary movie 1). The elastomer nanofibers significantly stabilised the co-cultures, permitting the generation of uniform co-cultures with minimal collapse for up to 2 weeks (figure 4(c), supplementary movie 2). Finally, nanofibers supported maturation of the neuromuscular co-cultures: After 2 weeks there were significantly more myofibers present in the cultures (figure 4(d)(i)) and these myofibers were significantly larger indicative of increased maturation (figure 4(d)(ii)). Furthermore, axonal outgrowth and NMJ size were also increased (figure 4(d)(iii), (iv)). Taken together these results show that oriented elastomer nanofibers support alignment, contraction, stability and maturation of hiPSC myofiber/motor neuron co-cultures in 96-well assay plates, and that the 2 weeks co-culture period allows the cellular components to connect and integrate into a functional neuromuscular circuit.

### 2.3. Automated HCI analysis of ALS-related neuromuscular phenotypes in 96-well plates

**Figure 5.**
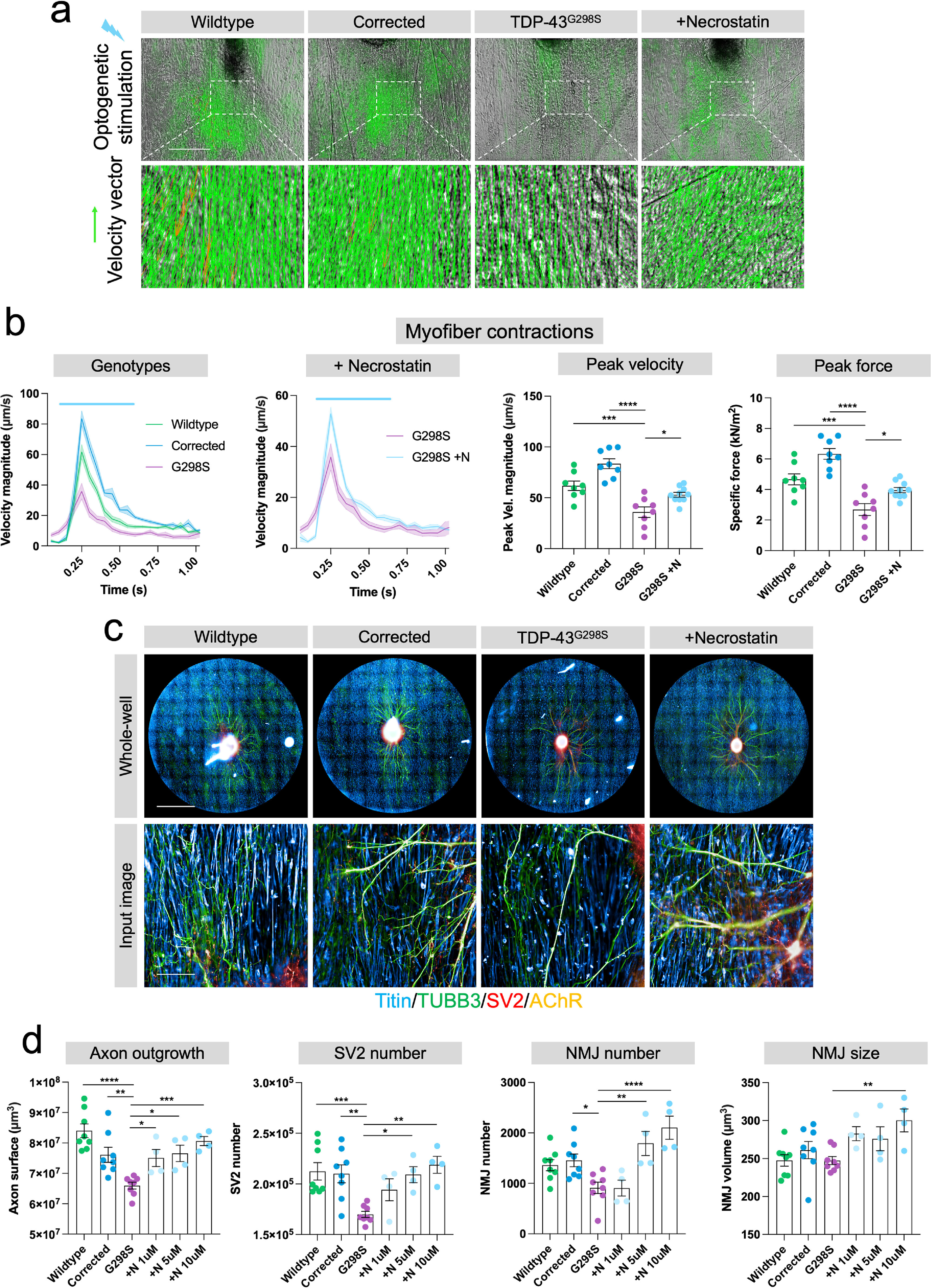
Automated HCI analysis of ALS-related neuromuscular phenotypes in 96-well plates after 2 weeks of culture. (a) PIV analysis of optogenetically evoked myofiber contractions in 96-well neuromuscular co-cultures containing wildtype, TDP-43^G298S^ CRISPR corrected, TDP-43^G298S^ and TDP-43^G298S^ + 10 *μ*M necrostatin-1 (*n* = 8 wells per condition) treated hiPSC-motor neurons. Scale bar = 200 *μ*m. (b) Quantification of myofiber contractions across different conditions. (ii) Contraction peaks for cultures containing wildtype, CRISPR corrected and TDP-43^G298S^ motor neurons. (ii) Contraction peaks comparing TDP-43^G298S^ cultures treated with and without necrostatin-1. (iii) Peak velocity values. (iv) Peak force values. (c) Whole-well and single field of view images taken using an automated operetta CLS HCI system. Scale bars = 2500 *μ*m, 200 *μ*m. (d) Automated quantification of (i) axon outgrowth, (ii) SV2 number, (iii) NMJ number and (iv) NMJ size across different conditions (*n* = 8 wells for wildtype, corrected and G298S; *n* = 4 wells for each concentration of necrostatin-1 in G298S co-cultures). One representative of three experiments is shown. Error bars represent the SEM, unpaired non-parametric t-test and one-way-ANOVA with Dunnet’s multiple comparisons used to ascertain statistical significance. *p < 0.05, **p < 0.01, ***p < 0.001, ****p < 0.0001.

Finally, we set out to recapitulate disease-specific neuromuscular phenotypes in the 96-well neuromuscular co-cultures, a key step toward making this platform a high-throughput tool for neuromuscular disease modelling and drug discovery. To achieve this, we generated co-cultures containing patient hiPSC-motor neurons harbouring an ALS-linked TDP-43^G298S^ mutation and used CRISPR-Cas9 gene editing to generate isogenic controls [25]. We found that co-cultures with TDP-43^G298S^ motor neurons showed significantly diminished optogenetically evoked myofiber contractions relative to wildtype and CRISPR corrected controls (figures 5(a) and (b); supplementary movies 3 and 4). We also observed that corrected controls showed significantly stronger contractions than WT controls (figure 5(b)(iii), (iv)). While both motor neuron populations carry two wildtype TDP-43 alleles, they are otherwise unrelated and will have many genetic differences which could impact on axon or NMJ function. Furthermore, we found that treatment with the RIPK1 inhibitor necrostatin-1 [26] partly restored myofiber contractility in the TDP-43^G298S^ co-cultures (figures 5(a) and (b); supplementary movie 5). In addition, we used a computational approach to convert velocity measurements of contractile output into estimates of contractile force (kN m^−2^) using the PIV analysis *x* + *y* vectors and elastic modulus data (figure 5(b)(iv)). Consistent with the velocity data, we found that TDP-43^G298S^ motor neuron co-cultures had significantly weaker contractions compared to wildtype and corrected cultures (figure 5(b)(i), (iii)), which again was significantly improved with necrostatin-1 treatment (figure 5(b)(ii), (iii)). Next, we employed the automated HCI analysis pipeline to assess axonal outgrowth, and NMJ number and morphology (figure 5(c)). We found that co-cultures containing TDP-43^G298S^ motor neurons showed a corresponding decrease in axonal outgrowth (figure 5(d)(i), pre-synaptic vesicle number (figure 5(d)(ii) and NMJ number (figure 5(d)(iii)), whereas NMJ size was not changed (figure 5(d)(iv)). In addition to this we observed that the RIPK1 inhibitor necrostatin-1 could partially rescue these phenotypes in a dose-dependent manner (figure 5(d)). Taken together, these results highlight the utility of this scalable neuromuscular co-culture platform for modelling neuromuscular disease phenotypes and screening for therapeutic compounds.

## 3. Discussion

In this study, we report the design and fabrication of a custom built 96-well plate with uniformly aligned elastomer nanofibers suspended in each well by electrospinning with a well-controlled moving spinneret. Using this technology, we were able to develop a hiPSC-derived neuromuscular disease model in a scalable format amenable to automated HCI analysis. In this model, biobased elastomer nanofiber scaffolds support long-term alignment, contraction, stability and maturation of neuromuscular co-cultures. Optogenetic stimulation of the hiPSC-motor neurons in the circuits elicited robust myofiber contractions, providing a functional readout of neuromuscular transmission. Since the assay was designed for multi-well imaging plates, it can be performed in a rapid and scalable manner. To our knowledge this is the first hiPSC neuromuscular disease model in which human neuromuscular disease phenotypes can be quantified automatically using HCI analysis—an important advance in the development of tools needed to better understand fundamental mechanisms of neuromuscular synapse physiology, and for screening small molecules and gene therapies aimed at treating neuromuscular diseases.

Previous models generated by ourselves and others have demonstrated the importance of stabilising contractile myofibers in order to generate mature and functional neuromuscular co-cultures [13–15, 19, 20]. However, the scalability of these previous systems has been limited, both in terms of the initial plating in small, compartmentalised sections of microdevices and/or on micropillars, and in terms of the tissue processing, imaging and analysis. We have achieved comparable and reproducible functional neuromuscular co-cultures in 96-well assay plates. Like the alternative models [14, 15], the myofiber construct we developed is a uniform 3D-layered structure suspended close to the base of each well, and the data is acquired as a z-stack of inverted confocal microscopy images. Other systems typically use a cylinder-like geometry suspended in the middle of each well and require some distance between the culture device base and the construct due to its suspension between pillars. In this configuration, directly imaging the live cylinder-like constructs at high resolution, especially in a 96-well plate, would become challenging using either an inverted or upright confocal microscope. In addition, the uniformity of each cylinder-like constructs could be difficult to control during the culture. In contrast, our myofiber construct, because of its consistent and uniform sheet-like geometry imposed by the nanofiber scaffold across each whole well, is located directly above the base and can be imaged at high resolution in its entirety. Whereas both geometries have analogues in human anatomy, such as the biceps brachii and the diaphragm muscles, respectively, a myofiber sheet facilitates image acquisition and analysis of structures like NMJs with automated HCI systems for drug screens. In terms of sensitivity, the two designs seem to be comparable: For example, myofiber contractions optogenetically induced by TDP-43-mutant motor neurons are about 2-fold smaller in amplitude than those elicited by wildtype/control motor neurons in both models [14] (figure 5(b)). However, micropillar-based systems, while lower-throughput, also have an advantage: measurement of pillar-deflection allows a more direct estimation of contraction force, whereas the PIV method we currently use for this purpose is indirect.

The initial plating is rapid since multi-channel pipettes can be used to seed the myoblasts as single cells. Furthermore, we developed a scalable approach to produce and plate neural spheroids by seeding dissociated motor neurons/astrocytes into non-adherent 96-well U-bottom plates to generate uniform spheroids. We then used a multi-channel pipette and a custom-built seeding mask to transfer these aggregates uniformly and consistently into the centre of the 96-well assay plates (figure 2(f)). In addition to this, the 96-well design allowed easy integration into existing HCI analysis systems such as the Perkin Elmer Operetta system used in this study. Thus, both the plating, imaging and analysis methods become more efficient, controllable, reproducible, and automated, making this system far more suitable for drug screening than previous neuromuscular co-culture systems that have been described in the literature.

Many previous studies have used hydrogels and bio-polymers to stabilise *in vitro* myofibers [19, 20, 27, 28], however in most cases these are rapidly degraded by the cells and it often takes time for endogenous ECM proteins to be synthesised and secreted, which affects muscle survival and maturation [28]. In other studies where myofibers are suspended in bundles between micropillars, compaction of the cells and non-transparency of the models makes it harder to carry out high power imaging and resolve individual myofiber and neuromuscular synaptic morphology. In this study we manufactured a thin layer of aligned synthetic elastomer nanofibers to act as a scaffold to facilitate myofiber alignment, contraction, long-term stability, and maturation (figure 4). These nanofibers do not degrade over the course of the cultures and do not interfere with the imaging of the cultures due to their transparency and absence of autofluorescence [24]. Since they are suspended and have matched elasticity with native skeletal muscle tissue, they facilitate contractile recoil of the myofiber sheet, predominantly along the axis of alignment (figures 4(a) and (b)) and in close proximity to the optical glass base, making it easy to carry out live imaging of myofiber contractions and fluorescent imaging of neuromuscular synapse morphology in the cultures at high resolution. Future iterations of this model could incorporate mechanosensitive piezoelectric nanofibers [29] coupled with 96-well multielectrode array technology to allow direct high-throughput electrical readouts of contractile force. Furthermore, the nanofibers also promoted axonal alignment, which is similar to native axonal morphology, opening the possibility of using this platform to carry out high-throughput axonal transport imaging assays in future studies, where long straight axons facilitate tracking of cargo.

We also further developed a simple, non-invasive, computational approach to estimate the contractile force transmitted in our neuromuscular model. Using velocity data derived from PIV analysis, we used Hooke’s laws to calculate an estimate of the stress vectors and subsequent force vectors in *X* and *Y* directions in the contracting myofiber-nanofiber constructs. Stress, or specific force (kN m^−2^), is a common metric used to define the force exerted by *in vitro* muscle constructs and is, therefore, useful data when comparing our system with existing systems in the literature. (figures 4(b) and 5(b)). While our approach currently does not permit direct force measurement, we have previously validated [24] that the specific force calculated using this method is within the range of values obtained from existing systems in the literature. While the accuracy of the estimation could be further calibrated experimentally, this computational pipeline can be used routinely alongside standard biochemical assays to assess relative functional responses to experimental stimuli, which is of particular interest to applications in longitudinal modelling and quantification of degenerative neuromuscular pathologies and early prediction of chemical drug responses.

As a proof-of-principle we used this platform to model ALS-related neuromuscular disease phenotypes by incorporating TDP-43^G29S^ motor neurons and CRISPR-corrected controls into the co-cultures. We selected TDP-43 mutant cells as a representative *in vitro* model for ALS, because aggregation of this protein is a common pathological hallmark of familial and sporadic ALS [30], and is seen in ca. 95% of all ALS cases. Likewise, necrostatin-1, the candidate drug tested in this study, is a compound known to be capable of rescuing ALS-like motor neuron degeneration *in vitro* [19, 26]. It inhibits the necroptosis programmed cell death pathway by blocking the activity of RIPK1. Candidate drugs which target the same pathway are currently being tested as ALS therapeutics in clinical trials [31]. We found that the ALS-linked TDP-43^G298S^ mutation caused a reduction in contractile strength, axon outgrowth and neuromuscular synapse number (figure 5), approximating symptoms of ALS seen in patients. We also showed that necrostatin-1 could rescue these phenotypes in a dose-dependent manner, demonstrating the feasibility of this co-culture platform for neuromuscular disease modelling and drug discovery. Since we enrich HB9^+^ motor neurons to ca. 95% purity by MACS prior to the assay, we can be confident that the contractile, axonal and synaptic phenotypes we observed are due to intrinsic defects in the motor neurons, unlike conventional mixed cultures, which show different efficiencies in hiPSC differentiation and variable proportions of motor neurons. This issue has been identified in previous studies on stem cell-derived models of ALS- and SMA-related neuromuscular disease phenotypes [14, 32]. Furthermore, the use of CRISPR-Cas9 genome editing to generate isogenic control lines allows us to attribute the phenotypes to a specific mutation, rather than to variation between unrelated hiPSC lines.

In conclusion, we have developed 96-well assay plates engineered with suspended, aligned elastomer nanofiber scaffolds with elasticity matched to skeletal muscle, to generate the first truly scalable 96-well human neuromuscular disease model. By combining hiPSC-derived neural and muscle cells, CRISPR-Cas9 genome editing, optogenetics, mask-position-controlled seeding and automated HCI analysis we were able to model ALS-related neuromuscular phenotypes and demonstrate a dose-dependent effect of the RIPK1 inhibitor, necrostatin-1, for rescuing disease phenotypes. We envisage that this will pave the way for future cost-effective and rapid small molecule and gene therapy screens aimed at developing new treatments for neuromuscular diseases.

## 4. Materials and methods

### 4.1. Polymer synthesis and electrospinning

Aligned electrospun nanofibers were fabricated from the biobased elastomer described in [24]. A polyester polyol, ethylene-diethylene-succinate (EDS), was synthesized by polycondensation reaction to yield a polyol with acid value: 0.39 mg KOH g^−1^ and hydroxyl value: 53.2 mgKOH g^−1^. Next, the EDS polyol was mixed with trans-cyclohexanechlorohydrinisobutyl-silsesquioxane (POSS) (Hybrid Plastics Inc., Hattiesburg, MS, USA) at 135 °C under nitrogen. The polyol blend and 4,4ʹ-diisocynato dicyclohexylmethane (H_12_MDI) were then reacted under nitrogen at 70–80 for 2 h in N-dimethylacetamide (DMAC), forming the pre-polymer. Finally, ethylenediamine in DMAC solution was added dropwise to the reaction to initiate chain extension, and 1-butanol was added to stop the chain extension reaction, such that a final polymer solution of 2% w/w POSS terminated polyurethane urea [P(EDS)UU-POSS] 18% w/w in DMAC was synthesised. A 10% w/w solution of P(EDS)UU-POSS in 1:1 acetone:dimethylformamide (DMF) was prepared for electrospinning. Aligned nanofibers were generated by electrostatic collection between two charged electrodes under a 20 kV electric field, inside a custom-built electrospinning set-up [24].

### 4.2. Custom 96-well plate manufacture

Elastomer nanofibers were incorporated into a 96-well format for high-throughput automated screening of neuromuscular co-cultures. To do this, black bottomless 96 well imaging plates (Greiner Bio-One, 6550000-06) were inverted and placed between two charged electrodes. The spinneret needle (14 G tip ground-to-flat needle) was connected to a linear slider controlled by a step-motor, which constantly displaced in the *x* direction (along the length of the microplate). This enabled an even distribution of the nanofibers across the base of the plate. All other parameters of the electrospinning set-up were used as previously described [24]. Nanofibers on each plate were electrospun for 30 min at a flow rate of 1 ml h^−1^ and a distance of 17 cm in a solution of 10% w/w in 1:1 mix of acetone:DMF. Membrane thickness was calculated to be 253 nm ± 103.6.

To render the plates water-tight, a 110 × 74 mm glass base (NEXTERION, 1535661) was attached beneath the nanofibers. A custom-made acrylic stamp was used to apply glue to the base of the plate while excluding the nanofiber-covered areas at the bottom of the wells (supplementary figure S5). Adhesives validated for biocompatibility standards ISO10993, and USP Class VI were utilized for the assembly of the plates. The stamp comprised a rectangle with 96 holes, fabricated from a laser-cut acrylic sheet. A thin layer of glue was applied to the stamp using a cell scraper. The plate was aligned and placed on top of the stamp, transferring the glue from the stamp to the plate. The holes in the stamp prevented the glue from contacting the nanofibers within the wells. The stamp was peeled off and replaced with the glass coverslip. The plates were cured at 60 °C for 24 h. The nanofiber plates were air plasma treated (Zepto Diener: 0.4 mBar, 20% power) for 2 min, and UV sterilised for 15 min. Prior to cell seeding, the nanofiber wells were incubated with 50 ul DMEM supplemented with GFR-Matrigel (1:100) for 2 h at RT. Control plates were assembled the same way as the nanofiber plates, except that the application of nanofibers by electrospinning was omitted. These plates were then plasma-treated, sterilized with UV-light and Matrigel-coated like the nanofiber plates.

### 4.3. SEM

Nanofiber sheets were visualized using a Zeiss focused ion beam SEM (XB1540). The nanofiber sheets were dried for 4 h at 50 °C, mounted on 13 mm SEM stubs, and gold sputter coated using a Quorum Technologies Q150R ES Gold Coater. The samples were imaged using a 10 kV beam current.

### 4.4. Cell culture and differentiation

The patient-derived hiPSC line harbouring the pathogenic *TDP-43^G298S^* mutation was provided by Agnes Nishimura and Christopher Shaw (King’s College London), and originated from the group of Siddarthan Chandran (The University of Edinburgh). The *TDP-43^G298S^* line was originally published in [33]. The donor provided written signed consent to donate their skin sample to derive iPSCs and their use was approved by the Ethics Committee from the King’s College Hospital, a national Medical Research Ethics Committee. The gene-corrected control iPSC line was generated by CRISPR-mediated recombination by the Lieberam group [25]. hiPSCs were differentiated in separate cultures into enriched populations of motor neurons and myoblasts according to the protocols developed in house. Briefly, patient-derived hiPSCs carrying the *TDP-43^G298S^* mutation, as well as isogenic control hiPSCs, were genetically engineered by knock-in into the *AAVS1* safe harbour locus to express the cell surface protein CD14 under the transcriptional regulation of the motor neuron-specific *Hb9* promoter. Mutant and control lines were further engineered to express the *CAG::ChR2-YFP* optogenetic actuator stably integrated into their genome by piggyBac-mediated transposition. Motor neurons were derived from these hiPSCs with extrinsic factors and then enriched to 90%–95% purity by MACS using the CD14 cell surface marker [25]. We engineered a doxycycline-inducible *PAX7* (iPAX7) myogenic determinant into the PAMV1 wildtype hiPSC line (www.hipsci.org/lines/#/lines/HPSI1013i-pamv_1) by knock-in into the *CLYBL* safe harbour locus, and then derived myoblasts from these hiPSCs by PAX7-mediated forward programming [24]. mESCs carrying a *GFAP::CD14* sortable marker and a *CAG::GDNF* neurotrophin transgene were differentiated into mixed neural cultures and then astrocytes were enriched by MACS according to [19]. 4 × 10^4^ iPAX7 myogenic progenitors were seeded into each well of the 96-well elastomer microplates in progenitor expansion medium (MegaCell DMEM (Sigma-Aldrich, M3942) with 5% fetal bovine serum (Sigma-Aldrich, F7524), 1% nonessential amino acid solution (Sigma-Aldrich, M7145), 55 *μ*M β-mercaptoethanol (Gibco, 21985023), 1% penicillin–streptomycin (Invitrogen, 15140-122), 2 *μ*g ml^−1^ doxycycline hyclate (Sigma-Aldrich, D9891), and 10 ng ml^−1^ bFGF (Sigma-Aldrich, F3685)). The following day, the medium was replaced with myofiber differentiation medium [25] (low-glucose DMEM (Gibco, 11885084) with 1% N2 supplement (Gibco, 17502001), 1% horse serum (Gibco, 26050070), and 1% penicillin–streptomycin). Neural spheroids were aggregated by plating 1 × 10^4^ hiPSC-motor neurons and 5 × 10^3^ mESC-astrocytes in Lipidure (Amsbio, CM5206)-coated U-bottom 96-well plates [19]. Three days post plating, single neural spheroids were transferred to the centre of each nanofiber well, using a custom-made (designed in AutoCAD—supplementary figure S6, supplementary file 1) 3D-printed (Raise3D Pro2 3D printer) seeding mask. The seeding mask served to allow consistent and rapid spheroid seeding at the centre of each well. The cultures were then maintained in a 1:1 mix of myofiber differentiation medium and ADFNB medium [19] (Advanced DMEM/F-12 medium (Gibco, 12634028) mixed 1:1 with Neurobasal medium (Gibco, 21103049), 1x Neurobrew-21 supplement (Miltenyi Biotec, 130-093-566), 1x N2 supplement (Gibco, 17502001), 2 mM L-glutamine (Gibco, 25030149), 1% penicillin–streptomycin, 55 *μ*M *β*-mercaptoethanol, 0.1% bovine serum albumin fraction V (Roche, 10735078001)). In some experiments, necrostatin-1 (Sigma-Aldrich, N9037) was added during the co-culture period at 1 *μ*M, 5 *μ*M or 10 *μ*M concentrations.

### 4.5. Immunofluorescence

In preparation for HCI analysis, co-cultured cells were fixed with 4% PFA for 15 min at RT and washed 3x in PBS. Subsequently cells were blocked and permeabilised in 3% BSA and 0.1% Triton X-100 in PBS for 1 h at RT. Cells were incubated overnight with primary antibodies: mouse IgM anti-Titin (DSHB—9D10), mouse IgG2a anti-TUBB3 (R&D Systems, MAB1195—Tuj1), Rat anti-AChR (DSHB—MAB35), mouse IgG1 anti-SV2 (DSHB—SV2) in blocking buffer at 4 °C. Cells were then washed 3x in 0.1% Triton X-100 in PBS and incubated with the secondary antibodies (Alexa fluor 405 anti-mouse IgM (Abcam, ab175662), Alexa fluor 488 anti-mouse IgG2a (Thermo Fisher Scientific, A-21131), Alexa fluor 555 anti-rat IgG (Thermo Fisher Scientific, A-21434), Alexa fluor 647 anti-mouse IgG1 (Thermo Fisher Scientific, A-21240)) at RT for 2 h. Cells were finally washed 3x in PBS. In addition, the following primary antibodies were used to characterise cellular phenotypes by immunocytochemistry: rabbit anti-ISL1 (Abcam, ab109517), rabbit anti-OLIG2 (Abcam, ab109186), mouse anti-MYOG (DSHB—F5D), mouse anti-myosin heavy chain (Invitrogen, 18-0714—MY32), mouse anti-PAX7 (DSHB—PAX7), mouse anti-M-cadherin (BD Biosciences, 611100).

### 4.6. HCI analysis

96-well neuromuscular co-cultures were imaged using a Perkin-Elmer Operetta CLS HCI analysis system and the Harmony 4.9 software. Plates were imaged in non-confocal mode using a 20x water objective. For each well, 69 fields were taken with a 20% overlap and 5 *Z*-planes at 2 *μ*m intervals with a binning of 2. For analysis using Harmony 4.9, advanced flatfield and brightfield correction was applied and stack processing was carried out using 3D analysis. ‘Find nuclei’ functions were used to generate initial masks for Titin, TUBB3, SV2 and AChR immunofluorescence. Threshold and splitting sensitivities were adjusted for each experiment depending on fluorescence intensity and background noise. The morphology and intensity calculator functions were used to derive morphological and fluorescence intensity parameters for all objects, which were then used to filter out background and non-specific staining. Mask filters were applied to SV2/AChR objects to derive objects that were co-localised to derive NMJ object values.

### 4.7. PIV and force calculations

Optogenetically-controlled contractions were recorded on an Olympus IX73 microscope fitted with a video camera. Light stimulation was carried out with an optical fibre–coupled 470 nm LED light source (Thorlabs, M470F3) controlled by a LED driver (Thorlabs, DC2200) [20]. Videos were captured at 20 frames per second and analysed by PIV using the PIVLab package [34] (https://pivlab.blogspot.com/) in Matlab (MathWorks). Local displacement of small interrogation regions (64/32/16 pixels, each with 50% overlap) was analysed between each frame of an image sequence (‘time resolved A + B, B + C′ format). Vectors were then validated by filtering out velocity values higher than 7 times the standard deviation. This enabled quantification of the velocity (*μ*m s^−1^) of myofiber contractions. To show that contractions were dependent on synaptic transmission at NMJs, 50 *μ*M of the AChR antagonist DTC (Sigma, 93750) was added to the co-cultures in control experiments.

The velocity (**ν**) vectors (**ν** *_x_* and **ν** *_y_*) were exported from PIVLab and used to calculate an estimate for specific force at peak contraction velocity, using Hooke’s laws. Displacement (**d**) vectors (**d** *_x_* and **d** *_y_*) were calculated using |**ν**| = |**d**|/*t*, and subsequently strain (*ϵ_x_* and *ϵ_y_*). Stress (**σ**) vectors were calculated using **σ** *_x_* = *ϵ_x_* E and **σ** *_y_* = *ϵ_y_E* where *E* = 30.5 kPa (average elastic modulus for cultured skeletal muscle reported in the literature). Force (**F**) vectors were estimated using **F** *_x_* = **σ** *_x_* A*_yz_* and **F** *_y_* = **σ** *_y_* A*_xz_*, where *A* was cross sectional-area. Specific force (kN m^−2^) = stress (kPa).

### 4.8. Directionality analysis

Nanofiber directionality was quantified using a fast Fourier transform in FIJI Software (https://fiji.sc/). The Directionality plugin was used to infer the preferred orientation of structures within the immunofluorescence image (based on Titin staining), generating a histogram of the number of structures in a given direction (0°–180°). Images that had a preferred orientation produced a histogram with a peak at that orientation. A larger peak infers more structures with the preferred orientation.

### 4.9. Statistical analysis

Data presentation and statistical analyses was performed in Prism 9 (Graphpad Software, San Diego, CA, USA, www.graphpad.com), Origin (Pro) Version 2021b and MATLAB. One-Way-ANOVA with Dunnet’s test for comparisons, and unpaired, non-parametric t-tests were used to infer statistically significant differences between samples and groups of samples and are specified for each figure. *P*-values <0.05 were deemed to be statistically significant and are denoted by*. **p < 0.01, ***p < 0.001, ****p < 0.0001. All values are represented as the mean ± SEM.

## Data Availability

All data that support the findings of this study are included within the article (and any supplementary files).
